# LPS-Induced Neuron Cell Apoptosis through TNF-α and Cytochrome c Expression in Dental Pulp

**DOI:** 10.1055/s-0043-1774329

**Published:** 2023-12-04

**Authors:** Galih Sampoerno, Eddo Supriyanto, Dian Agustin Wahjuningrum, Aghnia Alma Larasati, Dinda Ardiani, Meircurius Dwi Condro Surboyo, Anuj Bhardwaj, Ayver Atav Ates

**Affiliations:** 1 Department of Conservative Dentistry, Faculty of Dental Medicine, Univeristas Airlangga, Surabaya, Indonesia; 2 Conservative Dentistry Specialist Program, Faculty of Dental Medicine, Univeristas Airlangga, Surabaya, Indonesia; 3 Faculty of Dental Medicine, Universitas Airlangga, Surabaya, Indonesia; 4 Department of Oral Medicine, Faculty of Dental Medicine, Universitas Airlangga, Surabaya, Indonesia; 5 Department of Conservative Dentistry and Endodontic, Collage of Dental Science and Hospital, Rau, Indore, Madhya Pradesh, India; 6 Department of Endodontic, Faculty of Dentistry, Istinye University, Istanbul, Turkiye

**Keywords:** apoptosis, TNF-α, cytochrome c, dental pulp, neuron cell, immunology

## Abstract

**Objectives** Inflammation of the dental pulp tissue caused by bacteria, creating an immunology response of death of the dental pulp, is called apoptosis. The *Porphyromonas gingivalis* that cause apoptosis is lipopolysaccharide (LPS) through toll-like receptor (TLR) via two different mechanisms, intracellular and extracellular pathways. This study analyzed the role of LPS exposure of neuron cells, tumor necrosis factor-α (TNF-α), and cytochrome c (cyt-c) expression in the dental pulp to predict the possible mechanism of apoptosis.

**Materials and Methods** The lower tooth of Sprague Dawley rats was opened and exposed to LPS for 48 hours. Then the neuron cell analyzed histopathology using hematoxylin–eosin, whereas the TNF-α and cyt-c expression with indirect immunohistochemistry using a light microscope. The relationship between neuron cells with TNF-α and cyt-c was analyzed using stepwise regression linear analysis.

**Result** The LPS exposure showed a lower number of neuron cells and had a relationship with TNF-α expression but not with cyt-c, while compared with control, both TNF-α and cyt-c expression were higher in neuron cells.

**Conclusion** LPS exposure in dental pulp is possible to stimulate the apoptosis process through extracellular pathways marked by higher TNF-α expression.

## Introduction

Sustained caries can lead to the pulp chamber's opening, resulting in inflammation and pain. The inflammation in dental pulp is caused by bacteria products, like lipopolysaccharide (LPS) from *Porphyromonas gingivalis* , 1 through two recognizing the pattern recognition receptor and nucleotide-binding domain leucine-rich repeat-containing. 2 LPS are potent pathogen-associated molecular patterns recognized by toll-like receptor-4 (TLR4) that induces the production of proinflammatory cytokines, such as tumor necrosis factor-α (TNF-α), interleukin-6 (IL-6), and interleukin 1β (IL-1 β). 3 These cytokines cause inflammation in dental pulp nerve fiber that leads to neurodegeneration and destruction of the myelin sheath. 4 The degeneration can activate several factors and signaling pathways involved in the regulation of cell apoptosis. 5

Two main apoptotic pathways are caspase-independent and caspase-dependent. Apoptotic signaling of caspase-dependent pathways can occur intracellularly and extracellularly. The extracellular pathway is initiated by stimulating death receptors, whereas the intrinsic pathway is activated by releasing signaling factors from the mitochondria in cells. 6 In the death receptor pathway, the protein that acts as a receptor is the TNF-α receptor (TNFR) group. In contrast, the mitochondria will induce the intrinsic pathway by releasing cytochrome c (cyt-c) from the intermembrane of mitochondrial. Cyt-c is a heme protein that acts as an electron carrier in mitochondrial oxidative phosphorylation, stops the electron from cyt-c oxidase, exits the intermembrane, and binds to a cytoplasmic protein called Apaf-1, 7 and then activates caspase-9 and caspase-3. 8 In the dental pulp, caspase-9 is an important protein to induce apoptosis. 9 10

The TNF-α plays an important role in the extrinsic apoptosis pathway. Extrinsic apoptosis is initiated by binding specific ligands such as TNF-α, Fas ligand (FasL), and TNF-α-related apoptosis-inducing ligand to their corresponding receptors. TNF-α that binds to TNFR will produce adapter protein TRADD (TNFR-associated dead domain) with the recruitment of Fas associated with dead domain. This recruitment will activate caspase 8 and then activate caspase 3, which causes apoptosis.

The current research that dental pulp apoptosis stimulated by Bax and Bcl-2. 11 But there is no information about the exact dental pulp, especially in neuron cell apoptosis. This research is conducted to analyze the occurrence of neuron cells in dental pulp tissue after LPS induction through TNF-α and cyt c expression, also the apoptotic pathway that dominantly causes a decrease in the number of neuron cells due to inflammation.

## Materials and Methods

### Animals

Thirty-two male Sprague Dawley rats that met the inclusion criteria (in good health, body weight between 425-450 grams, age 20 weeks, and mandibular incisors completely erupted) were included in the study. Subjects were randomly divided into two groups: the control group and the LPS-induced group; each group consisted of 16 rats.

All the procedures conducted in this research had been reviewed and approved by the Health Research Ethical Clearance Commission, Faculty of Dental Medicine Airlangga University (Registration number 225/HRECC.FODM/V/2021).

### LPS

LPS, ultrapure lipopolysaccharide from *Porphyromonas gingivalis* —TLR4 ligand, was isolated from *Porphyromonas gingivalis* (InvivoGen, San Diego, Californian, United States).

### LPS-Induced Dental Pulp

Prior to the injection of LPS into the dental pulpal chamber of mandibular central incisors in rats, intraperitoneal anesthesia was administered using a combination of ketamine and xylazine. The pulp chamber was accessed using a high-speed handpiece (OM-T0307E, Pana-max NSK, Japan) fitted with a fissure bur (Dia-Burs, D14G007800 MANI, Kiyohara Industrial Park Utsunomiya, Tochigi, Japan). The incisors were cut at the transverse axis. Subsequently, preparation was carried out using a round bur until the pulp space was visually identified by a reddish appearance, followed by perforation using a dental explorer. Once the pulp chamber was exposed, 10 µl of LPS was injected, after which the cavity was sealed using glass ionomer cement. The control group, which did not receive LPS, was also sealed solely with glass ionomer cement.

Within the subsequent 48 hours, the animals were euthanized, and the mandibular samples were collected. The mandibular specimens were fixed in 4% paraformaldehyde and subsequently underwent decalcification using ethylenediaminetetraacetic acid over a period of one month. Following decalcification, the samples underwent processing to preparate for subsequent immunohistochemical staining.

### The Neuron Cell

The neuron cell was analyzed in dental pulp using hematoxylin–eosin under a light microscope (Nikon E100 LED binocular microscope, Nikon, New York, United States) at 1000× magnification in five different field analyses.

### The Expression of TNF-α and Cytochrome c

The expression of TNF-α and cyt-c was analyzed using indirect immunohistochemical staining. The antibody of TNF-α (mouse monoclonal antibody, Santa Cruz Biotechnology Inc, Texas, United States) and cyt-c (mouse monoclonal antibody, Santa Cruz Biotechnology Inc, Texas, United States) was used and counterstained with Mayer's hematoxylin. The TNF-α and cyt-c expression in the dental pulp tissue neuron cell were observed under a light microscope (Nikon E100 LED binocular microscope, Nikon, New York, United States) at 1000× magnification in five different field analyses.

### Data Analysis

The number of neuron cells, TNF-α expression, and cyt-c expression were analyzed with the Kolmogorov–Smirnov test to assess the data distribution and the Levene test for data homogeneity. Next, the independent t-test was performed to identify differences number of neuron cells, TNF-α expression, and cyt-c expression, between the control and LPS with a significant level of *p* -value less than 0.05. Later, the relationship between neuron cells and TNF-α expression and cyt-c expression was analyzed using the stepwise regression analysis test. All the test was performed using SPSS version 24 *(* IBM SPSS Statistic 24 for mac, New York, NY, United States) *.*

## Results

### The Neuron Cell in Dental Pulp

**Fig. 1:**
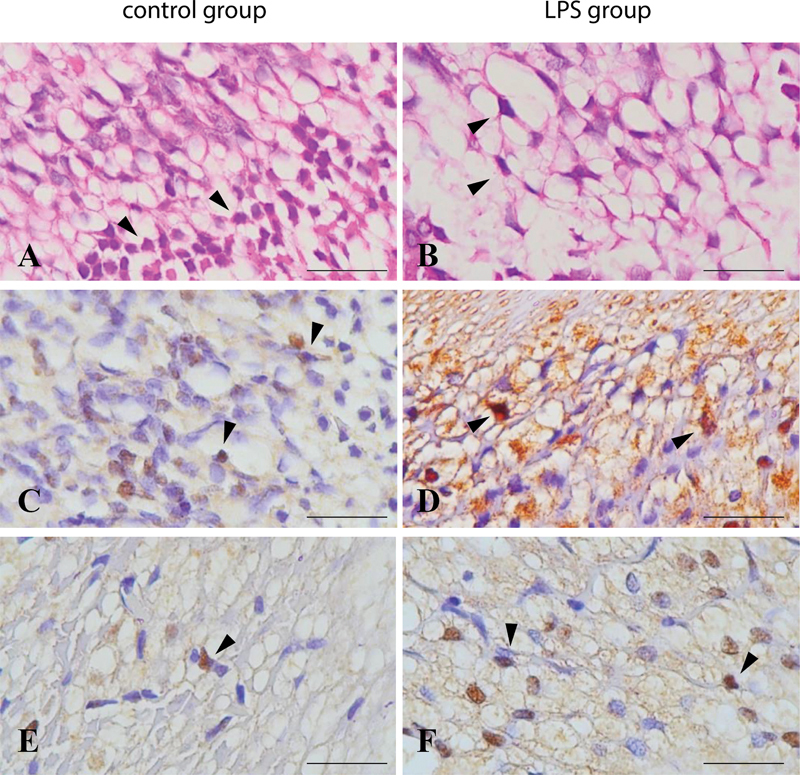
The histopathology of neuron cells in dental pulp ( **A** , **B** ) (hematoxylin and eosin; 400x magnification) and immunohistochemistry staining of tumor necrosis factor-α ( **C** , **D** ) and cytochrome c ( **E** , **F** ) (immunohistochemistry; 1000x magnification, scale bar 50 μm).

**Fig. 2:**
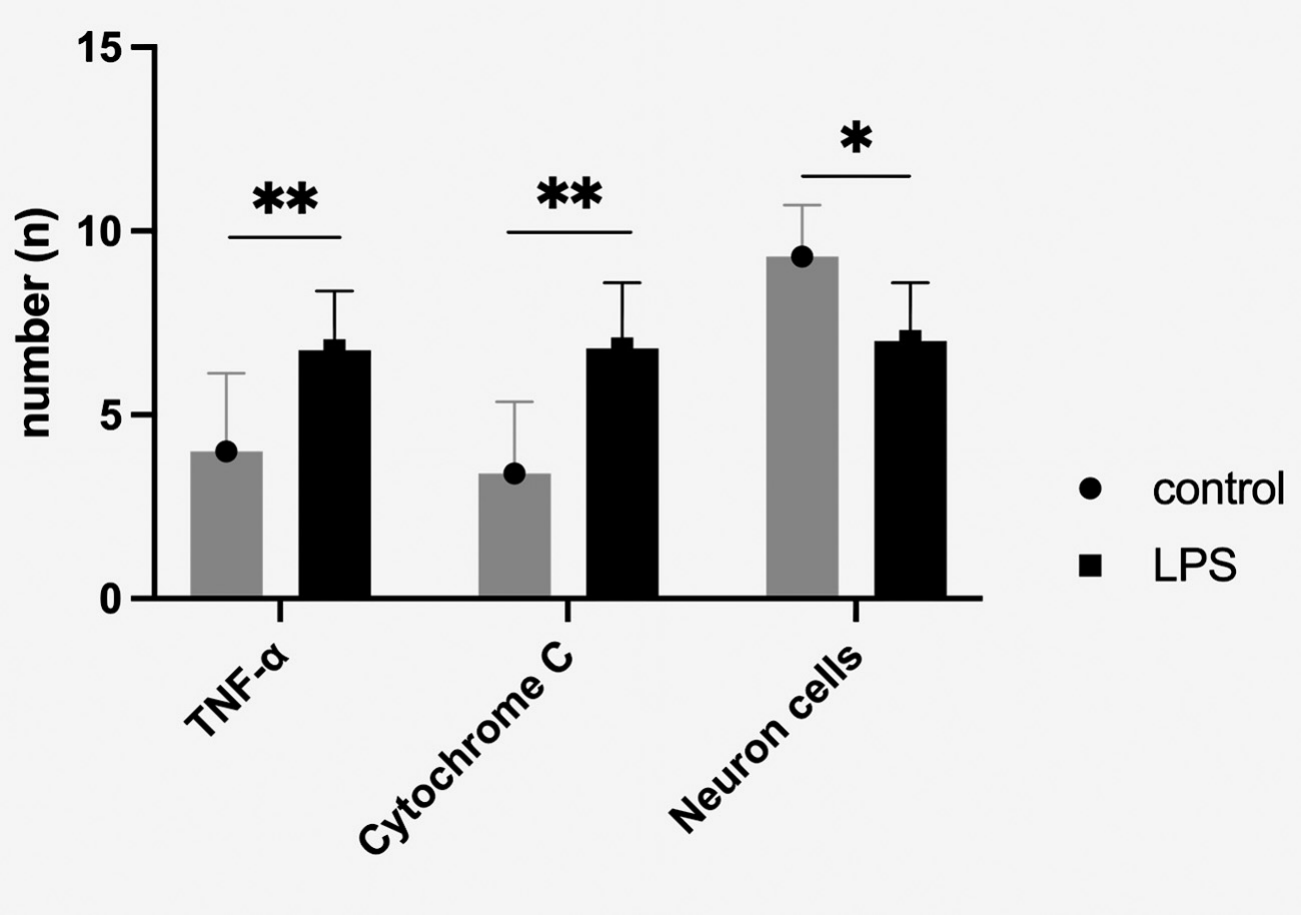
The number of neuron cells in dental pulps; tumor necrosis factor-α (TNF-α) and cytochrome c expressed by dental pulp neuron cells. * p<0.05, ** p<0.01. LPS, lipopolysaccharide.

The neuron cell apoptosis in histopathology analysis is shown in Fig. 1A, B . The LPS exposure showed a lower number of neuron cells than the control groups ( *p* < 0.05; Fig. 2 ).

### The Expression of TNF-α and Cytochrome c

The TNF-α and cyt-c expression in neuron cells is shown in Fig. 1C–F ). The LPS exposure showed a higher TNF-α expression in neuron cells than in the control groups ( *p* < 0.01; Fig. 2 ) **.** Similar to TNF-α expression, the LPS exposure also showed cyt-c expression in neuron cells ( *p* < 0.01; Fig. 2 ) **.**

### The Relationship between Neuron Cells, TNF-α, and Cytochrome c Expression

A negative correlation occurred between TNF-α expression and the number of neuron cells ( *p* = 0.038). Conversely, there is no correlation between cyt-c and neuron cells ( *p* = 0.075; Table 1 ).

**Table 1:** Mean, standard deviations, and *p* -value of independent t-test of TNF-α, cyt-c, and neuron cell expression

Variable	TNF-α		cyt-c	
	Coefficient value	*p* -Value	Coefficient value	*p* -Value
Neuron cell	−0.357	0.038*	0.304	0.075

Abbreviations: cyt-c, cytochrome c; TNF-α, tumor necrosis factor-α.

## Discussion

The LPS exposure to dental pulp showed a lower number of neuron cells than normal dental pulp. LPS exposure, induced inflammation, 12 imbalanced mitochondrial dynamics, and reduced cell differentiation without altering apoptosis and cell proliferation. 13 The lower number of neuron cells may be caused by an apoptosis process, through a different pathway—intracellular or extracellular pathways. The LPS is recognized by various TLR in the dental pulp; some research mention TLR1, TLR2, TLR6, and TLR4. 14 The recognition by TLR will trigger intracellular signaling to activate apoptosis.

**Fig. 3:**
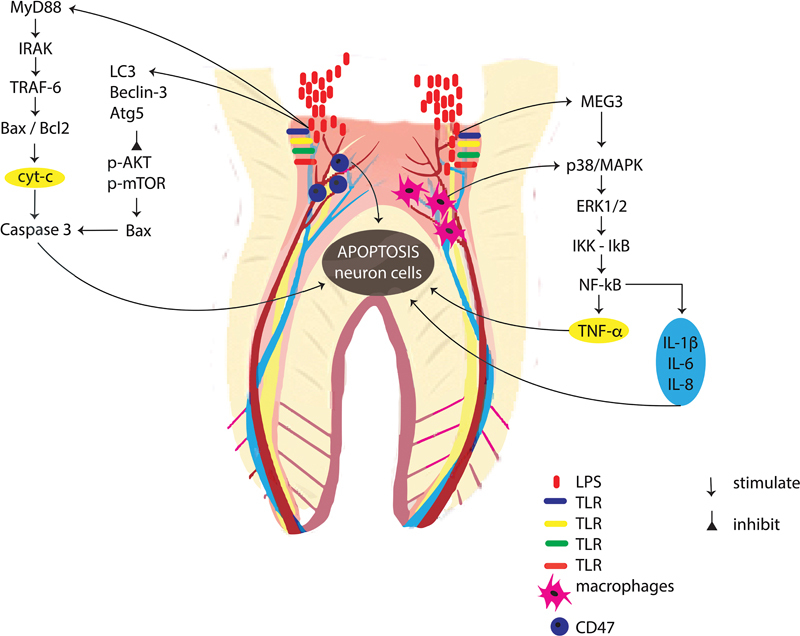
The possible mechanism of neuron cell apoptosis in dental pulp through tumor necrosis factor-α (TNF-α) and cytochrome c expression. IL-6, interleukin-6; IRAK, interleukin-1 receptor-associated kinase; LPS, lipopolysaccharide; MAPK, mitogen-activated protein kinase; MEG3, maternally expressed gene 3; NF-κB, nuclear factor kappa B; TLR, toll-like receptor; TRAF-6, TNF receptor-associated factor 6.

The first mechanism is through intracellular pathways, which are characterized by mitochondria damage, marked by an increase in the cyt-c expression. The exposure of LPS in dental pulp showed a higher cyt-c expression in dental pulp neuron cells. The TLR4 recognized will activate myeloid differentiation protein 88 (Myd88), which triggers intracellular signal transduction resulting in the activation of interleukin-1 receptor-associated kinase (IRAK), recruit TNF receptor-associated factor 6 (TRAF-6), and activates mitogen-activated protein kinase (MAPK). MAPK will activate the tumor suppressor protein p53, activate the Bax, 15 and then release the cyt-c into the cell cytoplasm. The LPS especially affected p53 activity via upregulation p16 expression through TLR-4. 16 Further, cyt-c binds to Apaf-1 to form a caspase recruitment domain. This process will activate caspase 9 and then activate procaspase-3 to become caspase 3. 17 The LPS exposure significantly increased the expression of XBP1, 15 LC3, Beclin1, and Atg5; decreased the expressions of phosphorylated protein kinase B (p-AKT) and phosphorylated mammalian target of repamycin (p-mTOR), and upregulated the expressions of caspase-3 and Bax. 18 The increase in caspase 3 and Bax, an effector caspase, causes neuron cells to undergo apoptosis or pyroptosis 19 ( Fig. 3 ). The LPS affected cyt-c release and decreased ATP production. 20

The second mechanism, through extracellular pathways, is marked by higher TNF-α expression in neuron cells in the dental pulp. The recognized LPS with TLR will activate the p38/MAPK, 21 extracellular signal-regulated kinase (ERK), 21 and c-jun N-terminal kinases (JNK) 22 and, in the final, activate the nuclear factor kappa B (NF-κB), 23 and produces proinflammatory cytokines such as TNF-α. 24 The other research also showed that the LPS signiﬁcantly upregulated maternally expressed gene 3, resulting in upregulating the secretion of TNF-α, IL-1β, 25 IL-6, IL-8, 23 and decrease in IL-10 21 through p38/MAPK signaling pathway. 26 In the cellular event, the response is provided by an increased number of neutrophils, macrophages and CD47. The CD47 plays a key role in the autophagy and apoptosis of odontoblasts 27 ( Fig. 3 ).

In this study, the number of neuron cells can be influenced by the cell death process through the extrinsic pathway by TNF-α and the intrinsic pathway by cyt-c expressions. From the relationship analysis, the number of neuron cells is affected by TNF-α. This study's results align with previous studies showing that LPS via the TLR4 pathway in the early phase of infection can activate MyD88, which triggers IRAK signal transduction. IRAK activation causes TRAF-6 to phosphorylate IKK inhibitors, which trigger and inhibit I-κB. I-κB is then deactivated, and thus NF-κB can be activated. NF-κB expression causes an increase in TNF-α expressing cells. 28 29

The dominancy of TNF-α showed that the extracellular pathways lead to apoptosis. Further studies need to confirm the other influence marker during extracellular and intracellular pathways-induced apoptosis in neuron cells.

## Conclusion

The LPS exposure in dental pulp is possible to stimulate the apoptosis process through extracellular pathways marked by higher TNF-α expression. But the other mechanism is questionable since the cyt-c, as an intracellular marker, found in higher numbers in neuron cells.
